# The use of patient-reported outcomes to detect adverse events in metastatic melanoma patients receiving immunotherapy: a randomized controlled pilot trial

**DOI:** 10.1186/s41687-020-00255-0

**Published:** 2020-10-30

**Authors:** Lærke K. Tolstrup, Lars Bastholt, Karin B. Dieperink, Sören Möller, Ann-Dorthe Zwisler, Helle Pappot

**Affiliations:** 1 Department of Oncology, Odense University Hospital, Odense, Denmark; 2 Department of Clinical Research, University of Southern Denmark, Odense, Denmark; 3 OPEN – Open Patient data Explorative Network, Odense University Hospital, Odense, Denmark; 4 The Danish Knowledge Centre for Rehabilitation and Palliative Care (REHPA), Nyborg, Denmark; 5 Department of Oncology, Copenhagen University Hospital, Copenhagen, Denmark

**Keywords:** RCT, Patient-reported outcomes, PRO, Melanoma, Adverse events, Toxicity, Immunotherapy, E-health, Patient involvement

## Abstract

**Background:**

A randomized controlled pilot trial was conducted to assess if melanoma patients treated with immunotherapy had the number of grade 3 or 4 adverse events during treatment reduced by 50% using a tailored electronic patient-reported outcomes tool in addition to standard toxicity monitoring compared to standard monitoring alone. Secondary endpoints were: if more AEs were reported in the intervention group, if there was a difference between the two groups in the number of telephone consultations, extra out-patient visits, number of days in the hospital, days in steroid treatment and the time patients experienced grade 2 or higher toxicity.

**Patients and methods:**

Melanoma patients receiving immunotherapy at the Department of Oncology, Odense University Hospital, Denmark participated. Standard care included assessment of AEs by a clinician before each treatment cycle using the Common Terminology Criteria for Adverse Events. In addition, patients randomized to the intervention reported their AEs weekly by an electronic PRO-tool based on the PRO-CTCAE platform.

**Results:**

One hundred forty-six melanoma patients were randomized. In this study, we did not detect a difference between the two groups in the number of grade 3 or 4 AEs (*P* = 0.983), in the overall number of AEs (*P* = 0.560) or in the time the patients in the two groups experienced grade 2 or higher toxicity (0.516). The number of phone contacts was significantly higher in the intervention group (*P* = 0.009) and there was a tendency towards patients in the intervention group having more extra visits (*P* = 0.156).

**Conclusion:**

It has been examined if the number of severe AEs for melanoma patients receiving immunotherapy could be reduced by involving the patients in the reporting of symptoms. The results do not justify the expansion of the pilot study into a regular phase III study with this particular set-up. However, a significant difference in the number of phone contacts was found as patients in the intervention group called more frequently, indicating that their attention to AEs was increased. Even though the use of an electronic PRO tool could not reduce the number of severe AEs in this melanoma population, a positive impact on other endpoints such as QoL, communication, or treatment-planning, cannot be excluded.

**Trial registration:**

Clinicaltrials.gov NCT03073031 Registered 8 March 2017, Retrospectively registered.

## Background

The number of people diagnosed with malignant melanoma worldwide has increased significantly during the last 50 years [1], which is in keeping with the development in Denmark [2]. Approximately 2300 new cases of melanoma are reported annually in Denmark, and more than 400 Danes are diagnosed with metastatic disease [3]. Despite the increase in incidence, survival has improved significantly due to new treatment modalities such as immune checkpoint inhibitors (CPIs) [4]. Furthermore, CPIs have resulted in significantly longer recurrence-free survival in the adjuvant setting [5]. It is well established that the toxicity profile of CPIs differs considerably from other cancer therapy strategies such as chemotherapy [6] and that immune-related adverse events (AEs) can be severe and, in some cases, life-threatening [7]. Since the introduction of CPIs, many trials have been carried out, which has not only improved survival significantly but also elucidated the adverse AEs related to CPIs [8–12]. Dealing with these AEs requires specific training of the caring physician and specialized nurses [4], and international guidelines to manage these toxicities have been developed [13]. It is well-known that early recognition may limit severity and duration [7]. Thus, it would be interesting to explore if it is possible to develop a clinical setup using an electronic solution including patient-reported outcomes to detect AEs at an earlier time-point before they turn into grade 3 or 4 AEs requiring hospitalizations, treatment with steroids and/or treatment discontinuation. In many oncology settings, toxicity-monitoring is carried out by a physician who assesses the patient before each treatment using the Common Terminology Criteria of Adverse Events (CTCAE) [14]. Apart from these scheduled visits, the patient usually may not have any contact with the hospital between treatments, i.e., typically for three to 4 weeks. The patients are informed about the specific toxicities which may arise. They are encouraged to contact the hospital in case of the occurrence of a symptom. However, some patients may still be reluctant to do so [15] either because they neglect their symptoms, worry that treatment may be stopped, or has not understood the importance of early detection. Accordingly, there is a risk that a symptom may go from mild to moderate/severe in this period. If patients become engaged in the reporting of symptoms on a more frequent basis, there is a presumption that AEs are discovered at an earlier time-point, enabling relevant treatment to be initiated and thereby avoiding major complications [16]. Studies suggest that using patient-reported outcomes (PROs) may result in improved communication, early relapse detection, optimized symptom monitoring, improved survival, and better quality of life (QoL) [17–19], particularly by the use of electronic devices [20]. However, it has not been examined if PROs in relation to symptom management for melanoma patients treated with immunotherapy may lead to earlier detection of symptoms resulting in a reduction in the number of severe AEs. Thus, based on current knowledge on AEs in melanoma patients receiving immunotherapy and PROs used in connection with symptom management, we hypothesized that self-reporting of AEs weekly direct from patients using a digital PRO system would be able to reduce the number of severe AEs during treatment compared to patients who get standard monitoring. To explore the above hypothesis, we designed a questionnaire from the PRO-CTCAE item library specifically tailored for melanoma patients receiving immunotherapy [21]. Following the development of the PRO tool, an open, randomized controlled pilot trial was conducted to assess if the number of grade 3 or 4 AEs during treatment could be reduced by 50% at 24 weeks follow up using the designed electronic PRO tool on patient self-reporting, in addition to standard toxicity monitoring compared to standard monitoring alone. If the preliminary assessment was positive and implementation viable, the plan was to proceed with a national, multi-center phase three study including 2 other Danish sites. Because this pilot trial was relatively large, we intended to include the collected data in the results of the larger randomized controlled trial (RCT). This would be possible if the nature of the adjustments and improvements made as a result of in the pilot trial did not alter the study protocol substantively [22].

## Materials and methods

### Setting

At the Department of Oncology, Odense University Hospital (OUH), approximately 100 patients with metastatic melanoma are treated each year. Recruitment took place at OUH between January 2017 and May 2019. Patients were introduced to the study when they were informed about treatment with a CPI. Before the first treatment, the patients were contacted by telephone and asked to give oral and written informed consent.

### Design

This study cites an open, randomized controlled pilot trial, PROMelanoma (ClinicalTrials.gov NCT03073031). The consort checklist for the Reporting of Patient-Reported Outcomes in Randomized Trials was followed. Patients were randomly assigned and allocated sequentially numbered containers in a 1:1 ratio using the computer software program Open Patient data Explorative Network [23] to one of the following groups: standard toxicity assessment performed by a physician using the CTCAE before each treatment cycle or standard toxicity assessment performed by a physician before treatment supplemented by weekly web-based electronic reporting at home. Randomization was stratified according to treatment (anti-CTLA-4 vs. anti-PD1 or anti-CTLA-4/anti-PD1 in combination) and disease status (treatment for metastatic disease vs. adjuvant therapy after surgery for metastatic disease).

### Standard care

Patients had their adverse events assessed by a clinician using the CTCAE before each treatment cycle. The patients were informed orally and in writing about the treatment and the toxicities, which may occur. The importance of contacting the hospital in cases of the occurrence of new symptoms was also emphasized to the patients. In Denmark, an algorithm exists, in alignment with international guidelines, which describes in detail how specific AEs should be handled [24].

### Intervention

**Fig. 1:**
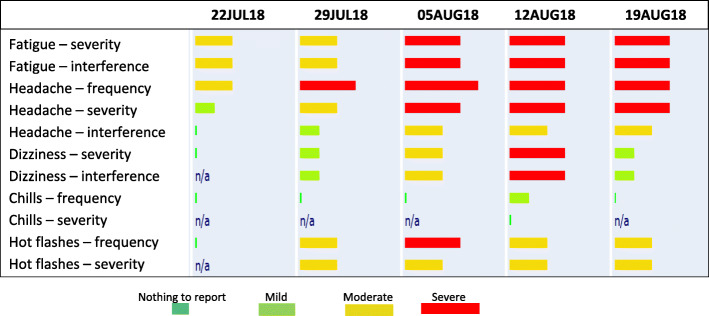
Example of patient reporting available to clinicians

In addition to standard care, patients in the intervention group received a tablet computer with a sim-card to ensure all patients could participate in the web-based evaluation. Moreover, they were trained in the self-reporting of symptoms. Baseline registration was made at the clinic. The software platform AmbuFlex [25] was used for patient reporting. Studies demonstrate that the vast majority of AEs occur within 24 weeks of treatment [26, 27]. Accordingly, the patients reported weekly for a maximum of 24 weeks. The patients did not receive a reminder, but were asked, when introduced to the intervention, to report their symptoms on a fixed weekday, making reporting easier to remember. If the patients stopped treatment due to toxicity or disease progression before this time-point, toxicity-monitoring would take place for 30 days after the last dose of immunotherapy or until the initiation of other anti-neoplastic therapy. As soon as the patients reported a mild or higher AE, an alert was triggered for the majority of AEs telling the patient to contact the hospital. The patients were instructed to contact the usual nurses´ line like other patients. Thus, there was not an on call nurse or physician specific for this study. The alert was triggered for 24 out of the 29 items included in the questionnaire. No alerts were triggered for *fatigue, skin dryness, hair loss, decreased appetite,* and *taste changes* because these symptoms were not at risk of becoming severe overnight. A clinician did not routinely monitor the patient reports. When the patients came for their scheduled appointment in the out-patient clinic, the physician would log into the system to see the patient reporting and discuss it with the patient. Figure 1 shows what the reporting looked like for the clinician. A bar attached to each symptom appeared green (no/mild), yellow (moderate), or red (severe) depending on the frequency and severity of the symptom and how much it affected daily activities. The differing widths of the red bars indicate whether the AE was severe or very severe.

### Participants

Eligible patients had unresectable stage III or stage IV disease and were scheduled to receive a CPI either as monotherapy or in combination as first, second or third line therapy. Patients treated with a CPI as monotherapy in adjuvant settings could also be included. Other eligibility criteria included the age of at least 18 years; be able to read and understand Danish; be willing and able to comply with the completion of an electronic PRO-questionnaire on symptoms and required Qol questionnaires*.* Baseline characteristics such as age, gender, performance status, disease stage, and experiences with electronic devices, were collected.

### Method for patient reporting

The American National Cancer Institute (NCI) has developed standardized definitions for AEs – The Common Criteria for adverse events to describe the severity of organ toxicity for patients receiving cancer therapy [14]. The system consists of 780 adverse events, and in the beginning, it was primarily used in clinical trials. Today, however, it is also used in routine cancer treatment. In order to enhance patient involvement, the NCI has developed the CTCAE scoring system for toxicity-monitoring into a tool appropriate for patient self-reporting [28]. An item-library of 78 items have been found appropriate for self-monitoring and constitutes now the PRO-CTCAE [29]. The PRO-CTCAE item library has been translated and validated in a Danish context [30]. Because existing questionnaires may not adequately capture the toxicities unique to CPIs [6], this item bank was chosen for this study, making it possible to design a questionnaire fitted for melanoma patients receiving immunotherapy. A thorough item-selection process was carried out, which has resulted in a questionnaire consisting of 29 items [21]. Weekly reporting was chosen since this is the preferred recall period in PRO-CTCAE questionnaires [31].

### Statistical considerations

It was expected that 140 patients could be recruited over a two-year period. By including 140 patients, a 50% reduction in the proportion of patients experiencing severe AEs could be detected with a one-sided significance level of 0.2 and power of 0.64 for an unadjusted comparison of two proportions. This power was considered acceptable since the trial was a pilot study evaluating a new health technology [32]. Baseline characteristics and AEs by randomization groups were reported as counts and proportions. Moreover, we compare the number of AEs, phone contacts, and extra visits to the outpatient clinic by Poisson regression, respectively, negative binomial regression, in case of detected overdispersion. We compare the total duration of grade 2 or higher AEs, duration of hospital stay and duration of steroid treatment by Wilcoxon rank-sum test, and display the total length of grade 2 or higher AEs a Kaplan-Meier curve. All analyses were carried out in Stata 15.0 [33].

### Primary outcome

The importance of early detection of AEs during treatment with immunotherapy to avoid them from becoming severe has been underlined repeatedly in the literature [7, 16, 34]. Similarly, studies have demonstrated that PROs can be useful in the early detection and monitoring of symptoms [35, 36]. Thus, using PRO to ensure early detection seemed logical for this patient population. We aimed at achieving a large improvement by implementing PRO in this population as a change in workflow should be meaningful to both patients and clinicians, as well as valuable to the health care system. Accordingly, we decided on examining if the number of grade 3 or 4 AEs assessed by the CTCAE could be reduced by 50% by having patients more actively involved in the reporting of symptoms.

### Secondary endpoints

To explore if more AEs were reported in the intervention group, if there was a difference between the two groups when it comes to number of telephone consultations and extra out-patient visits, if the time patients experience grade 2 or higher toxicity differs in the two groups and if there is a difference in the number of days in hospital and if the number of days in steroid treatment differs.

## Results

### Patients and treatments

**Fig. 2:**
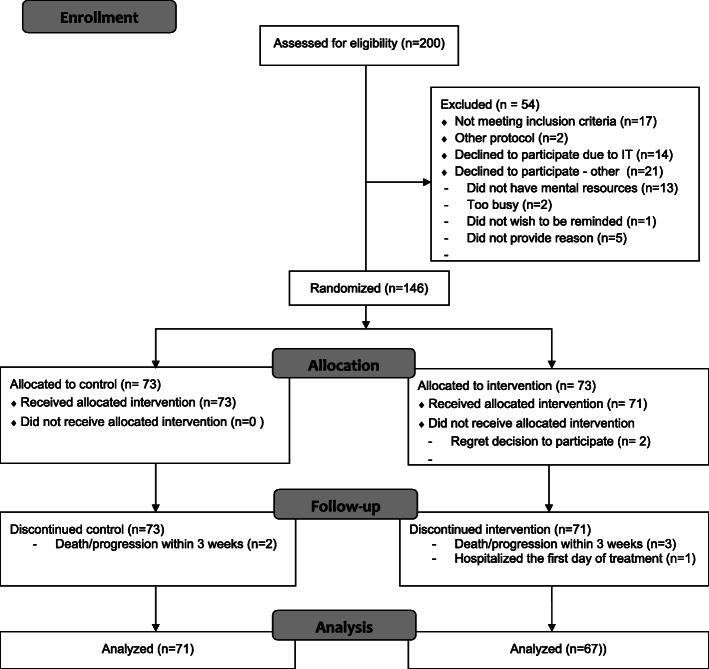
Consort diagram of inclusion process

Two hundred patients were screened for the trial, and 181 patients were considered eligible. Among these patients, 146 were randomized to the trial between January 2017 and May 2019. Thirty-five patients declined to participate (Fig. 2). Among the 35 patients who declined randomization, 14 patients gave IT-related reasons, whereas 13 patients believed AE reporting was too demanding. The median age of the patients who declined to participate due to IT was 78 years, compared to 66 years in the randomized group. Two patients withdrew their consent to participate, and six patients were excluded within the first 3 weeks after randomization due to rapid disease progression.

The majority of patients (67%) received Pembrolizumab or Nivolumab as monotherapy (Table 1). The 24 patients who received adjuvant therapy were all treated with Nivolumab. Only seven patients (5%) received Ipilimumab. Thus, less than one third of the patients (28%) received the combination therapy. The last recruited patient made the final report at the end of October 2019. The patients reported AEs between 4 and 25 times. The average number of reporting was 17 times. The vast majority of the patients (*n* = 52/78%) adhered to the intervention by reporting their symptoms on a weekly basis either throughout the whole period (*n* = 31) or until disease progression or intolerable toxicity (*n* = 21). Thus, only 15 (22%) of the 67 patients did not adhere completely to the weekly reporting, but reported more sporadically.

**Table 1:** Patient characteristics at baseline in the randomized trial

	Control *N* = 73 (%)	Intervention *N* = 73 (%)	*P*-values
Random assignment 73			
Ipilimunab	3 (4)	4 (6)	0.956
Pembrolizumab	36 (49)	38 (52)	
Nivolumab	13 (18)	11 (15)	
Ipilimumab+Nivolumab	21 (29)	20 (28)	
Age			
Median (range)	66 (32; 83)	66 (34; 87)	0.619
Sex			
Male	43 (59)	35 (48)	0.184
Female	30 (41)	38 (52)	
ECOG Performance status			
0	52 (72)	49 (69)	0.582
1	19 (26)	19 (27)	
2	1 (1)	3 (4)	
Disease stage			
Stage III	12 (16)	10 (14)	0.818
Stage IV	61 (84)	63 (86)	
Line of therapy			
Adjuvant	13 (18)	11 (15)	0.841
1st line	52 (71)	52 (71)	
2nd line	6 (8)	6 (8)	
3rd line	2 (3)	4 (5)	
Lactate dehydrogenase			
Normal	51 (76)	46 (69)	0.334
Elevated	16 (24)	21 (31)	
BRAF status			
Mutated	31 (42)	32 (44)	0.710
Wild type	30 (41)	27 (37)	
Unknown	12 (16)	14 (19)	
Experience with electronic devices			
None	0 (0)	3 (6)	0.232
A little	16 (38)	15 (32)	
A lot	26 (62)	29 (62)	
Educational level			
Primary/lower sec.	16 (23)	19 (28)	0.731
Upper secondary	2 (3)	3 (5)	
Short cycle tertiary	15 (22)	18 (27)	
Bachelor	30 (43)	23 (34)	
Master	6 (9)	4 (6)	

Comparisons of baseline characteristics in the two groups show that there are no significant differences between the intervention and the control group. Baseline characteristics are shown in Table 1. The median age in both groups was 66 years (range: 32–87). 53% of the participants were male, and 47% female. The majority of patients (69%) had performance status 0. Three of the included patients (6%) reported that they had no computer experience.

### Primary and secondary outcomes

As for the number of severe AEs (grades 3 and 4), the primary outcome, there was no significant difference between the two groups (*P* = 0.983), which is also the fact for the overall number of reported AEs (*P* = 0.560). A sub-analysis comparing the number of grade 3 and 4 AEs corresponding to the PRO items showed no difference either (Table 2). However, only eight events related to a PRO-item were reported in the control arm and six in the intervention arm. Thus, approximately one-third of the severe AEs that occurred were the same as the symptoms that the patients were asked about in the PRO-CTCAE questionnaire. In addition, more than one-third of the grade 3 and 4 AEs were elevated liver enzymes, creating few symptoms for the patients to report upon.

**Table 2:** Overview of treatment-related events, contacts, days in hospital and days in steroid treatment

	Control n	Intervention n	*P*-value
All treatment-related events			
Any grade	202	202	0.560
Grade 3 or 4	20	19	0.983
Events related to PRO-items			
Any grade	129	124	0.779
Grade 3 or 4	8	6	0.622
Treatment-related contacts			
Phone calls	102	163	0.009
Extra visits/ER visits)	31	44	0.156
Hospitalizations			
Numbers	32	50	0.119
Days (accumulated)	131	221	0.101
Steroid treatment			
Numbers	28	41	0.629
Days (accumulated)	714	1133	0.004

**Fig. 3:**
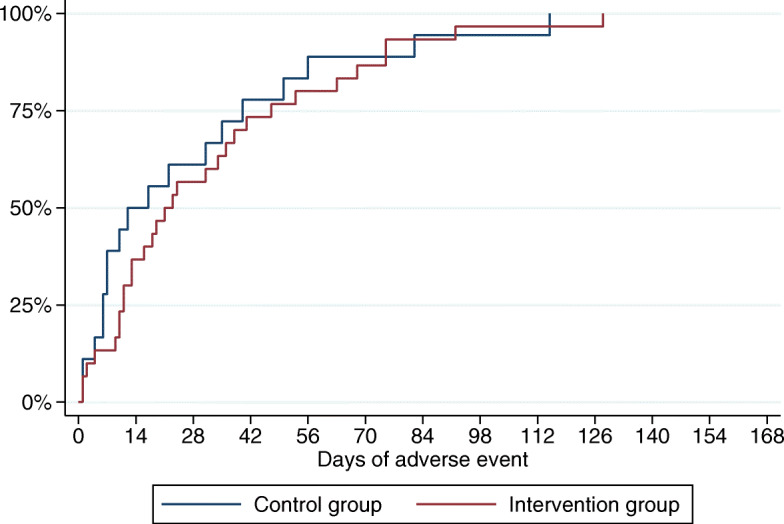
Duration of grade 2 or higher adverse events

The overall number of patients who experienced a grade 3 or 4 event was 58% for the combination therapy and 13% for patients who received anti-PD1 as monotherapy. There was no significant difference in the time the patients in the two groups experienced grade 2 or higher toxicity (0.516) either (Fig. 3). There was a significant difference in the number of phone calls to the hospital, as patients in the intervention group called more frequently (*P* = 0.009). However, 13 patients (19%) represent almost half of the phone calls (47%) in the intervention group, which means that a minority of patients called frequently. There was also a tendency towards patients in the intervention group having more extra visits (including emergency room visits) (*P* = 0.156), which correlates to the higher number of extra phone calls. A significant difference was found in the number of days patients received steroid treatment. Patients in the intervention group had more days on steroid treatment (*P* = 0.004). However, there was not a significant difference in the actual number of steroid treatments (*P* = 629). When it comes to the number of days in the hospital, there was a tendency (*P* = 0.101) that patients in the intervention group had more days in the hospital compared to patients in the control group, which corresponds to the number of hospitalizations (*P* = 0.119). However, in total, only a small number of patients received steroids or were admitted to the hospital.

## Discussion

This randomized controlled pilot trial aimed to compare the number of severe (grade 3 and 4) AEs developed during standard toxicity monitoring versus standard toxicity-monitoring plus weekly patient reporting for melanoma patients receiving immunotherapy. In this study, we did not detect a difference between the two groups, and we did not see a difference in the overall number of AEs either. Patients in the intervention group called significantly more often, indicating that they reacted on the triggered alerts and were thus more aware of their symptoms compared to patients in the control group.

There may be several explanations as to why we did not detect a difference in the number of grade 3 and 4 AEs. Although the need for early detection is underlined again and again in the literature, our study demonstrates that a relatively large group of AEs cannot be detected at an early point, using patient self-reporting systems. For example, elevated liver enzymes, which constituted approximately one-third of the severe AEs in this trial, are usually asymptomatic [37]. They can only be detected by blood samples which are carried out before each treatment cycle according to existing guidelines [24]. Another reason may be that the overall attention to AEs was increased based on the information all patients received about the clinical trial, perhaps leading to an unanticipated reduction in the number of severe AEs in the control group resulting in the two groups experiencing similar improvements [38]. Patients in the control group had been introduced to the study before randomization, which might have increased their desire to contact the hospital unscheduled. No design of a clinical trial could have avoided this. However, the number of patients who developed severe AEs aligns with the numbers found in the literature [5, 10, 39–42], which indicates that the risk of bias is negligible. Furthermore, because immunotherapy is still relatively new as a cancer treatment strategy, there may also be a general tendency to be more aware of the toxicity profile. Oncologists and oncology nurses in Denmark specialized in treating melanoma patients receiving CPIs are very attuned to potential severe AEs which may occur. Consequently, patients are well informed on how to react in case new symptoms occur, and there may not be much to improve because of the high standard of routine care. Bruin et al. argue in a non-cancer study that the level of routine care to a great extent determines how much improvement in behavior change can be achieved [43]. Had the study been carried out in another setting with a poorer quality of care, results may have been different. Also, a relatively small proportion of the patients (less than one third) received the combination therapy, where the risk of developing severe AEs is much higher compared to monotherapy. Had we included only patients who had received the combination, a greater number of severe AEs would have occurred, making our dataset larger.

Regarding the patient population we have examined, there was also a built-in risk that the less technologically avid patients may also be the ones who declined to participate. More than 75% of the patients who declined to participate did so either due to lack of computer skills or because they believed it would be too demanding. The median age of the patients who declined due to IT-related reasons was 78 years compared to 66 years for the patients who were included. Only three of the patients included in the study reported that they had no computer experience beforehand. These numbers indicate that technology was a barrier when trying to recruit older/computer-naive patients to our RCT. This result is in line with Fiteni et al., who argue that patients who are computer-naive may be excluded from this kind of intervention [20]. These patients may also be the ones who would benefit the most from the intervention because they may be less likely to contact the hospital unscheduled. According to Basch et al., patients with no IT skills may have weaker communication skills and therefore benefit more from a structured set-up [18]. If our study had had a more complex set-up with an oncology nurse contacting the patients when the alert was triggered, the patients reluctant to call might have been reached, and AEs might have been detected at an earlier time point. Other studies suggest that this pro-active approach may be the way forward [17, 18].

We did see a significant difference in the number of phone calls between the two groups, which demonstrates that the attention to AEs was increased, as was the intention of the study. The threshold (when an alert was triggered as a result of patient reporting) for contacting the hospital may have been set too low. Too many alerts may have been triggered, resulting in too many irrelevant phone calls/extra visits to the hospital. Moreover, the patients who were already inclined to call the hospital may call even more. The fact that 13 patients represented almost half of the phone calls supports this argument. Patients who were already reluctant to call the hospital may, on the other hand, continue to be hesitant and disregard the alert. To what extent the patients actually did react on alerts will be examined in a future study. There was also a tendency towards patients in the intervention group having more days in the hospital and more hospitalizations. In relation to steroid treatment, a significantly higher number of patients in the intervention arm received steroids due to an AE. However, there was not a significant difference in the number of steroid treatments, indicating that a small number of patients may have had long periods on steroid treatment. The number of patients who had received steroids, or had been admitted to the hospital, however, was low, and the results should therefore be interpreted with caution. Moreover, it does seem highly unlikely that the electronic reporting AEs would put patients in the intervention group in a poorer position compared to the control group.

Patients and clinicians were also asked about their experiences with the intervention through a survey and interviews [44]. Overall, patients and clinicians agreed that the attention to AEs was increased and that the patients were better prepared for the consultation when they came to the out-patient clinic. Moreover, the patients believed that the electronic questionnaire was easy to access and fill out. Thus, in terms of clinician and patient satisfaction, the study did make a difference for the included patients. QoL-data was also collected during the trial using the FACT-M and the EQ-5D-5L questionnaires. It will be elucidated if the high patient and clinician satisfaction is also reflected in the patients´ QoL when QoL-data from this patient population is analyzed and reported. Other PRO-studies have demonstrated an improved QoL for patients in the intervention group [18] and it is extremely important that this potential benefit is not overlooked.

### Strengths and limitations

It is an obvious strength that a randomized controlled trial was carried out to evaluate the primary endpoint. Furthermore, the chosen PRO-questionnaire was specifically designed for patients receiving immunotherapy. It may be a limitation that it was a single-center study and a pilot study with a small sample size. The fact that a minority of the randomized patients received combination therapy contributed further to this, making the number of patients at risk of developing severe AEs relatively small. In addition, only approximately a third of the severe AEs were related to PRO items, which is clearly a challenge to the interpretation of the primary outcome. Moreover, one third of the severe AEs were laboratory abnormalities, which should have been taken into account at the beginning. Furthermore, less technologically avid patients may have declined to participate, and the set-up may have been too simple, because patient reports were not monitored in real-time by a clinician. It could also be argued that this pilot study may be larger than needed to determine if large effects were possible. However, it made sense, as it was our intention to include the collected data in the results of a larger RCT. Moreover, we planned to evaluate the intervention using a survey, which required an adequate sample size.

## Conclusion

In this RCT it was examined if the number of grade 3 and 4 AEs for melanoma patients receiving immunotherapy with CPIs could be reduced by actively involving the patients in the reporting of symptoms using a PRO tool. The results we have presented regarding our primary aim, do not justify the expansion of the pilot trial into a regular phase III study. However, addressing the limitations, when designing a subsequent trial, may result in a different outcome. Also, a significant difference in the number of phone calls was found as patients in the intervention group called more frequently indication that patients´ attention to AEs was increased and even though the use of an electronic PRO tool could not reduce the number of severe AEs in this melanoma population, a positive impact on other endpoints such as QoL, communication, or treatment-planning, cannot be excluded.

## Data Availability

Informed consent forms, electronic patient reporting and patient data are stored at the Department of Oncology, Odense University Hospital, DK.

## Abbreviations

CPIs: Checkpoint inhibitors
AEs: Adverse events
CTCAE: Common Terminology Criteria of Adverse Events
PROs: Patient-reported outcomes
QoL: Quality of life
OUH: Odense University Hospital (OUH)
CTLA-4: Cytotoxic T-lymphocyte-associated protein 4
PD-1: Programmed cell death protein 1
NCI: National Cancer Institute
